# Supplementary material for: Manipulation of anisotropic Zhang-Rice exciton in NiPS3 by magnetic field
Source: Nat Commun. 2024 Sep 7;15:7841. doi: 10.1038/s41467-024-52220-w (PMC11380663; doi:10.1038/s41467-024-52220-w)
Supplement: Supplementary file 1 — Supplementary Information [file 41467_2024_52220_MOESM1_ESM.pdf]

Supplementary Information

Manipulation of anisotropic Zhang-Rice exciton in $NiPS_3$ by magnetic field

Feilong Song[1#], Yanpei Lv[1,2#], Yu-Jia Sun[1,2], Simin Pang[1, 2], Haonan Chang[1, 2], Shan Guan[1], Jia-Min Lai[1, 2], Xu-Jie Wang[3], Bang Wu[3], Chengyong Hu[3], Zhiliang Yuan[3], Jun Zhang[1,2]*

[1]State Key Laboratory of Superlattices and Microstructures, Institute of Semiconductors, Chinese Academy of Sciences, Beijing 100083, China

[2]Center of Materials Science and Optoelectronics Engineering, University of Chinese Academy of Sciences, Beijing 100049, China

[3]Beijing Academy of Quantum Information Science, Beijing 100193, China

*Email: zhangjwill@semi.ac.cn

[#]These authors contributed equally to this work.

Contents:

Supplementary note 1. Experimental details.

Supplementary note 2. Data analysis.

Supplementary note 3. Monte Carlo simulations.

Supplementary note 4. The explanation of the polarization direction and spin-polarization alignment.

Supplementary note 5. Comments on the linewidth and lifetime of the ZRE.

Supplementary Figure S1. Experimental setup of the polarization-resolved magneto-optical measurements.

Supplementary Figure S2. Temperature-dependent PL spectra of ZRE from $NiPS_3$ thin flakes.

Supplementary Figure S3. Power-dependent and sample position-dependent PL spectra of ZRE.

Supplementary Figure S4. Zeeman splitting of the spin-correlated excitons in sample_1.

Supplementary Figure S5. Monte Carlo simulations of the spin orientation with XY-type Heisenberg Hamiltonian.

Supplementary Figure S6. Polarization, magnetic moment, and Zeeman effect of ZRE.

Supplementary Figure S7. Sample images and polarization-resolved PL of ZRE of sample_2 when the magnetic field is nearly along the **b**-axis.

Supplementary Figure S8. The PL spectra of ZRE in sample_1 measured at 4 K with varying the magnetic field $\mu_0\mathbf{H}$.

Supplementary Figure S9. Polarization-resolved PL intensity of ZRE under an external magnetic field perpendicular to **a**-axis.

Supplementary Figure S10. The PL spectra of ZRE in sample_2 with flipping the magnetic field $\mu_0\mathbf{H}$.

Supplementary Figure S11. Magnetic field-dependent parameter *A* of sample_1 at 4 K and sample_2 at 1.6 K.

Supplementary Figure S12. The lifetime and linewidth of the ZRE.

Supplementary Figure S13. The temperature and power dependence of ZRE FWHM.

Supplementary Table S1. The reports about the linewidth and lifetime of the ZRE.

Supplementary note 1. Experimental details

Two samples were measured with almost the same experimental designs, and the results obtained at a magnetic field below 9 T for both samples are consistent. Both samples were mechanically exploited from the same bulk sample. There were only two differences in the measurements of sample_1 and sample_2:

1, Each time the sample orientation was changed during the measurement of sample_1, the sample went from 4 K to room temperature and then back down to 4 K; Sample_2 was maintained at 1.6 K during the angle-resolved magneto-optical experiments.

2, Sample_1 was measured under a field below 9 T while the magnetic field applied in the measurements of sample_2 could reach 12 T.

The **a**-axis is determined with the Zeeman splitting and polarization of Zhang-Rice exciton (ZRE). Below the Néel temperature, the direction of the Néel vector of $NiPS_3$ is along the **a**-axis of the crystal, as confirmed by neutron scattering and X-ray diffraction experiments. Thus, we determine the **a**-axis by ascertaining the direction of the Néel vector at 0 T with the application of an in-plane magnetic field. The optical setup of our angle-resolved and polarization-resolved measurements is depicted in the **Supplementary Figure S1**. We rotated the sample using an in-situ rotator while applying an in-plane magnetic field to the sample. In the angle-resolved PL experiments conducted from 0 T to 9 T, we observed that the Zeeman splitting of ZRE reached its maximum and exhibited high linearity in a specific orientation of the sample, as shown in the leftmost panel in **Supplementary Figure S8**. Conversely, in the sample orientation perpendicular to this, the Zeeman splitting was almost negligible, as shown in the rightmost panel in **Supplementary Figure S8**. As the Zeeman splitting is proportional to the magnetic field component along the Néel vector, we attributed these two sample orientations as corresponding to the cases when the Néel vector remained parallel and perpendicular to the external magnetic field while increasing the magnetic field, respectively. Additionally, at the sample orientation corresponding to the maximum Zeeman splitting, we increased the magnetic field further to observe the spin-flop phase transition, as shown in **Figure Supplementary Figure S10 a**, which is highly sensitive to the angle between the magnetic field and the **a**-axis and can assist in more accurately determining the **a**-axis. A similar angle-resolved measurement has been used to determine the Néel vector at 0 T in the research of MnF_2 [1].

By vertically polarizing the laser using a polarizer, we determined the polarization of the PL or laser when it propagated to the sample or to the spectrometer by introducing an analyzer or employing analysis based on the principle of light polarization. We measured the ZRE polarization while rotating the sample at 0 T and found that the polarization could synchronously follow the sample rotation, indicating that we can conveniently determine the **a**-axis through the polarization of ZRE at 0 T. To establish the relationship between the ZRE polarization and the **a**-axis at 0 T, we conducted the experiments as follows. According to the methods discussed above, we first rotated the sample to the orientation in which the **a**-axis is parallel with the magnetic field. Then, we measured the polarization of vertical polarized laser at 0 T and compared it with the

polarization of ZRE. As a result, we then determined **a**-axis with the polarization of ZRE at 0 T.

The θ, representing the angle between magnetic field and **a**-axis of sample, was determined as follows. The only directly measured angles in our experiments are the relative sample orientations, which were determined with a camera and we change the orientation with a step of about 15° with an error of no more than 3°. $\theta = 0^{\circ}$ was identified with the angle-resolved measurements as mentioned above. The errors of the angles θ are determined by the resolutions of the camera, spectrometer, and the exciton peaks.

Supplementary note 2. Data analysis

The Zeeman splitting of the ZRE in NiPS3 is fitted with the formulae:

$$\tan(2\varphi) = \frac{\sin(2\theta)}{\cos(2\theta) - \frac{H^2}{H_c^2}}, \tag{1}$$

$$\Delta E = 2\Delta g \mu_B \mu_0 H \cos(2\varphi). \tag{2}$$

The definition of φ is shown in the **Figure 3 c**, while the ΔE, μ_B and $\mu_0 H$ represent the Zeeman splitting energy, the Bohr magnetic moment and the magnetic indu.

The fitting procedure is detailed as follows. Since the only directly measured angle in our experiments is the relative angle of sample, as shown in **Supplementary Figure S7**, we should define a reference angle θ_0 as the angle where we measure the maximum of the Zeeman splitting. The $\mu_0\mathbf{H}$-dependent Zeeman splitting can be fitted with setting θ_0 and $\mu_0 H_c$ as the adjusted parameters. With setting $\theta_0 = 0^{\circ}$, the critical field $\mu_0 H_c$ was fitted as shown in **Supplementary Figure S11**. With the fitted $\mu_0 H_c$, the Zeeman splitting was fitted by adjusting the θ at each magnetic field orientation. If the fitted θ approximately formed an arithmetic progression with a common difference of 15° and the θ_0 is approximately equal to 0°,this fitting process was reasonable. The data in the main text **Figure 3** and **Supplementary Figure S8 c** was fitted with this procedure.

Supplementary note 3. Monte Carlo simulations

We have performed Monte Carlo simulations to calculate the physical quantities in 1 layer of honeycomb lattice containing 16 × 16 honeycombs. The system of spins is described by the Hamiltonian[2]:

$$\mathcal{H} = \frac{1}{2}\sum_{\alpha=a,b,c}\left[\sum_{\langle i,j\rangle} J_{1\alpha} S_{i\alpha} S_{j\alpha} + \sum_{\langle\langle i,j\rangle\rangle} J_{2\alpha} S_{i\alpha} S_{j\alpha} + \sum_{\langle\langle\langle i,j\rangle\rangle\rangle} J_{3\alpha} S_{3\alpha} S_{3\alpha}\right] - D\sum_i (S_i^x)^2 + g\mu_B\mu_0 \mathbf{H}\cdot\mathbf{S}. \tag{3}$$

where single, double, and triple angular brackets in the sums denote the nearest, next-nearest, third-nearest neighbors on the same plane, respectively. $J_{1\alpha}$, $J_{2\alpha}$ and $J_{3\alpha}$ are the nearest, next-nearest, third-nearest coupling parameters along α direction in the frames of the crystal axis, respectively. D is the easy-axis single-ion anisotropy. In our simulation, we set the parameters along different directions to be equal and $[J_{1\alpha}, J_{2\alpha}, J_{3\alpha}, D]$ to be $[-1.9, 0.1, 1.9, 0.08]$ eV. The temperature was selected as 1.6 K.

As shown in the **Supplementary Figure S5 a**, the unit cell was selected as a rectangle including four magnetic ions labelled with 1-4. As shown in the **Supplementary Figure S5 b-g**, only the simulated results of the spin component on the position 1 and 3 are shown because that the simulated results on the position 2 (4) is the same as those on position 1 (3). As shown in **Supplementary Figure S5 h-j**, the angle between the Néel vector and the magnetic field was selected as the average of the angle between antiparallel spins, with which we simulated the behavior of the Zeeman splitting using the formula (S2), as shown in the **Supplementary Figure S5 k**. These fit results are consistent with the experimental data as shown in the **Figure 3.**

Supplementary note 4. The explanation of the polarization direction and spin-polarization alignment.

In the Zeeman effect in metal ions transitions, as shown in **Supplementary Figure S6 a**, linearly polarized light collected parallel to the magnetic field is referred to as π light[3,4]. Here π light corresponds to transitions with unchanged magnetic quantum numbers, meaning the angular momentum along the magnetic field direction remains the same during the transition. As a result, the propagating direction of the emitted photons is perpendicular to the magnetic field. The probability of photon propagation in all directions within the plane perpendicular to the magnetic field is equal, which cancels out the electric field component perpendicular to the magnetic field, leaving only the electric field component parallel to the angular momentum, resulting in the polarization of π light being parallel to the magnetic field. Even if the magnetic field is rotated, the π light rotates accordingly and always keeps parallel to the magnetic field.

In our experiment, as shown in **Supplementary Figure S12 b** and **c**, the orientation of local magnetic moments was identified using anisotropic Zeeman splitting, and it was found that the polarization of PL remained parallel to the local magnetic moments in all orientations. As a direct result of our polarization experiment, the PL is π light and the total magnetic quantum number remains unchanged during the radiative transition of spin-correlated excitons. The spin-correlated exciton corresponds to a spin-flip process[5,6], implying that the component of orbital angular momentum in the spin direction should change to preserve total angular momentum. This orbital change is allowed by that both Zhang-Rice singlets and triplets compose by a *d*-orbital hole and a *p*-orbital hole[7], excluding the potential *d-d* transition mechanism.

Within $NiPS_3$, the Zhang-Rice singlet is located in an octahedron consisting of a Ni atom and six S atoms, where a hole occupies a *d* orbital of the Ni atom, and a portion of the *p* orbitals of the six S atoms in the octahedron contribute to the hole[5]. According to the theory proposed by Zhang and Rice[8], the ZRS orbitals form bonding states with strong overlap between the ligands and the transition metal[5]. In ZRS, the symmetry of the hole on the ligands matches that of the hole on the transition metal, leading to the consideration of the hole on the S atoms as a *d* orbital hole. On the other hand, the ZRT orbitals form antibonding states, resulting in relatively independent ligand and transition metal holes, with the hole on the S atoms maintaining its original *p* orbital characteristics[9]. Even though there is orbital quenching, the *p* orbitals located outside the easy plane can still contribute to orbital angular momentum[9]. Therefore, the

transition from ZRS to ZRT causes a change in orbital angular momentum since it corresponds to the transition from a *d*-orbital hole to a *p*-orbital hole.

The orbital change helps explain why the ZRE has a shorter lifetime compared to optical transitions in other 3d ions, such as CuB_2O_4[10], MnF_2[11], and Cr_2O_3[12], which arise from *d-d* transitions that are parity-forbidden. In contrast, the change in orbital angular momentum means ZRE is allowed by the dipole transitions selection rule, leading to larger transition matrix element and then rapider radiation transition rate and shorter lifetime[13]. Additionally, the involvement of p orbitals of S introduces *J-J* coupling[14,15], avoiding spin-forbidden limit and increasing the radiation transition rate compared with spin flip process in d-d transition.

As shown in **Supplementary Figure S12 d**, this orbital behavior complements our understanding of spin reorientation transition, where the magnetic field reorientate the local magnetic moments $\boldsymbol{\mu}$ to a new stable direction in $NiPS_3$. Under this field, ZRT and ZRS possess different gyromagnetic ratios g, resulting in anisotropic Zeeman splitting. The total local magnetic moments originate from the spin angular momentum and orbital angular momentum. Since the orientation of orbital angular momentum is related to the orientation of orbital wave function, the magnetic field simultaneously drives the rotation of both spin and orbital. Due to orbital reduction effect[16,17], the g-factor contribution from orbital is reduced and the g factor of ZRS is small, while the main contribution to g factor of ZRT comes from the spin and is big. As a simplification, it is assumed that the spin-flip occurs in the central *d* orbitals, while the orbital change occurs in the *p* orbitals and the *p* hole transition from ${}^{2}D_{5/2}$ *to* ${}^{2}P_{3/2}$. The *p-d* orbital hybridization could reduce the orbital g factor and the orbital reduction factor is denoted as *k*. The magnetic moment of ZRS could be calculated to be $0.8k_{ZRS}\mu_B$ considering the orbital contribution from ${}^{2}D_{5/2}$ state in the *L-S* coupling scheme[17]. The magnetic moment of ZRT mostly originates from the total spin ($2\mu_B$) and the orbital contribution ($0.6k_{ZRT}\mu_B$) of ${}^{2}P_{3/2}$. The difference of local magnetic moment between ZRS and ZRT could be calculated to be $(2+0.8k_{ZRS}-0.6k_{ZRT})\mu_B$. As discussed above the *p-d* orbital hybridization is weaker in ZRT, k_{ZRS} *is* smaller than k_{ZRT}. This is consistent with the experimentally measured splitting energy of $3.9\mu_B B$ when a magnetic field is parallel with the local magnetic moment. The accurate g-factor for the ZRT in $NiPS_3$ can be obtained through electron paramagnetic resonance measurements. Our Zeeman result could help obtain the g-factor and k-factor of the excited state and then help specifically determine the orbital configuration of the ZRS.

In summary, the polarization direction and spin-polarization alignment of ZRE suggest that the change in orbital compensates for the change in angular momentum during the spin-flip transition from ZRS to ZRT. Under the conservation of angular momentum, the rotation of exciton polarization originates from the rotation of local magnetic moments, corresponding to synchronous rotations of both spin and orbital of ZRE. The change in orbital angular momentum could be attributed to the alteration in the exchange symmetry of orbital accompanying the spin-flip. The orbital change,

allowing the selection rule of electric dipole transition and then obtaining rapider radiation transition rate, could explain the short lifetime of the spin-correlated excitons. The orbital change could exclude the potential *d-d* transition interpretation and then support the Zhang-Rice interpretation of this exciton.

Supplementary note 5. Comments on the linewidth and lifetime of the ZRE.

To make the discussion more reliable, we measured the lifetime of the ZRE, as shown in **Supplementary Figure S12 b**. In **Supplementary Table S1**, we also listed the reported lifetime and the linewidth of the exciton emission in $NiPS_3$. All samples synthesized by the chemical vapor transport (CVT) method have a short lifetime from 10 ps to 40 ps and narrow linewidth from 260 to 770 μeV, but the sample synthesized by liquid phase exfoliation (LQE) method has a longer lifetime of 1 ns and broader linewidth of 1.7 meV[18-21]. In order to measure the lifetime, we re-prepared the samples using the mechanical exfoliation method. The sample shows ~17 ps lifetime and ~482 μeV linewidth from **Supplementary Figure S12**, showing a comparable crystal quality with other reports that use CVT samples.

The experimentally measured linewidth and lifetime of ZRE correspond to the decay of different physical quantities. The mentioned ZRE lifetime in the main text corresponds to the exciton population decay time (T_1), which includes contributions from both radiative lifetime (T_{rad}) and non-radiative lifetime ($T_{\mathrm{non-rad}}$)[22,23]: $\frac{1}{T_1} = \frac{1}{T_{\mathrm{rad}}} + \frac{1}{T_{\mathrm{non-rad}}}$. The linewidth (Γ) of the exciton peak corresponds to the $\frac{\hbar}{T_2}$, which is related to the overall phase relaxation time T_2 of the exciton[22,23]. The Γ is not only from the exciton population decay rate ($\sim 1/2T_1$), but also from the pure dephasing rate ($\sim 1/T_2^*$) and inhomogeneous broadening (Γ_{inhom}). The pure dephasing of the exciton includes contributions from scattering with phonons, other electron excitations, and defects[23]. The relationship among them is given by[22,23]: $\Gamma = \frac{\hbar}{T_2} \sim \frac{\hbar}{2T_1} + \frac{\hbar}{T_2^*} + \Gamma_{\mathrm{inhom}}$. The population decay and pure dephasing processes ($\frac{\hbar}{2T_1} + \frac{\hbar}{T_2^*}$) contribute to homogeneous broadening.

At low temperature, the *Γ* of ZRE in references [19-21] and this work is generally larger than $\frac{\hbar}{2T_1}$, indicating that the mechanisms dominating the ZRE linewidth are pure dephasing or inhomogeneous broadening[19]. The linewidth of ZRE in LQE samples is larger than in CVT-grown samples, which can be attributed to the increased disorder in LQE leading to greater inhomogeneous broadening and disorder-related pure dephasing [18]. The temperature and laser power-dependent PL help us to understand the pure dephasing contributions to line broadening associated with phonons and other electronic excitations[23,24]. As shown in **Supplementary Figure S13**, the linewidth of ZRE remains almost unchanged when varying temperature below 20 K and varying laser power at 4 K. This suggests that at 4 K, phonons or other electron excitations hardly contribute to the linewidth broadening of ZRE. The narrower linewidth of ZRE

suggests that defect-related broadening of ZRE is smaller, which could originate from its spin-correlated nature and BEC-like coherence [5].

For the ZRE lifetime, the effect of inhomogeneous broadening mainly increases disorder and then increases the non-radiative transition rate in principle [2]. However, the observation that increasing disorder extends the ZRE lifetime [5] suggests that disorder suppresses radiative transition and the radiative transition dominates the ZRE lifetime. Compared with other excitons, the shorter lifetime of ZRE originates from the faster radiative transition. Our polarization experiments reveal that the ZRE transition process follows the orbital selection rules, which, to some extent, explains the higher radiative transition rate (i.e., shorter lifetime) of ZRE.

In summary, the narrow linewidth of the ZRE could originate from small inhomogeneous broadening and small defect-related pure dephasing due to its spin-correlated nature and BEC-like coherence. The short lifetime of ZRE is due to its faster radiative transition rate, while the inhomogeneous broadening effect inhibits radiative transitions, thereby extending the ZRE lifetime in some samples such as liquid phase exfoliated samples.

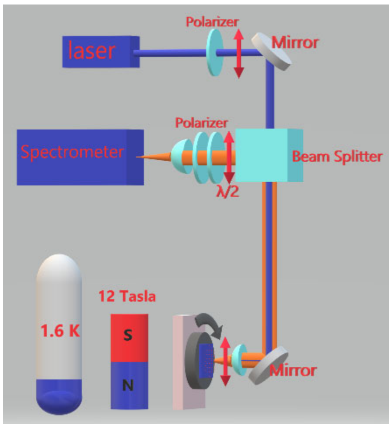

Supplementary Figure S1. Experimental setup of the polarization-resolved magneto-optical measurements. In the measurement, we use a 532 nm or 633 nm laser to excite the $NiPS_3$ thin flakes. A 50× long working distance objective is used to focus the laser on the sample and collect the PL signal. To apply the magnetic field in the Voigt geometry, the sample is placed vertically in the magnetic cell with the surface parallel to the direction of the applied magnetic field. A mirror is placed between objective and the optical window (does not shown in the figure) at an angle of 45° to change the optical path by 90°。 The magnetic field ranges from 0 T to 12 T and the temperature is about 1.6 K. The PL signal is reflected to the spectrometer by the beam splitter. In front of the spectrometer, we put a polarizer and a half-wave plate to detect the polarization of the PL signal. We use a piezoelectric in-situ rotator to rotate the sample at low temperature and a high magnetic field.

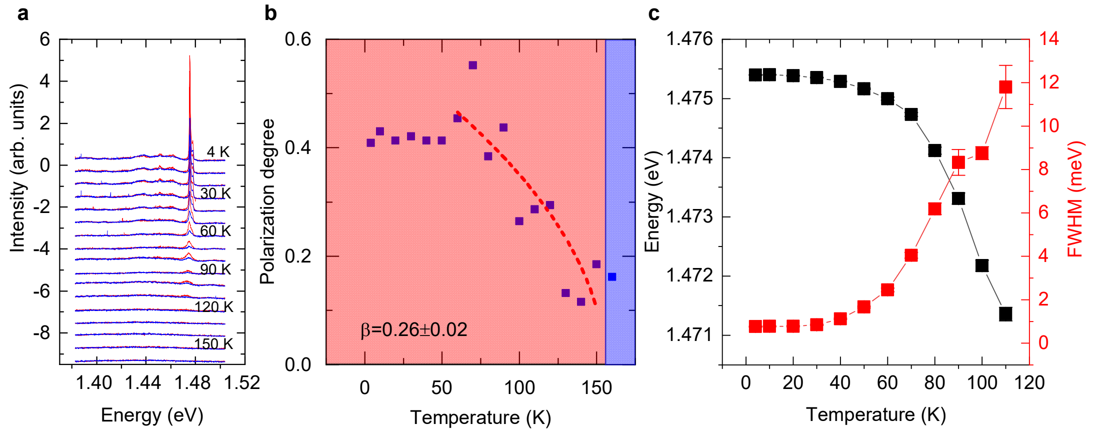

Supplementary Figure S2. Temperature-dependent PL spectra of ZRE from $NiPS_3$ thin flakes. **a**, PL spectra as a function of the temperature. The blue curves are the PL collected perpendicularly to the **a**-axis, while the red curves are the PL collected parallel to the *a*-axis. The temperature ranges from 4 K to 160 K. The PL signals disappear above 120 K, which is approximately 30 K below the Néel temperature T_N. **b**, The degree of polarization (ρ) as a function of temperature T. The dashed line denotes the fitted curve, $\rho(T) \propto \left|1 - \frac{T}{T_N}\right|^{2\beta_{PL}}$, where $\beta_{PL} = 0.26 \pm 0.02$, in consistent with 2D XY-type spin systems[20]. **c**, The energy and the full width at half maxima (FWHM) of the X_1 exciton extracted with a Lorentz peak function. The error bars are defined as the fitting errors of the peak positions and FWHM of X_1 exciton peaks in **a**.

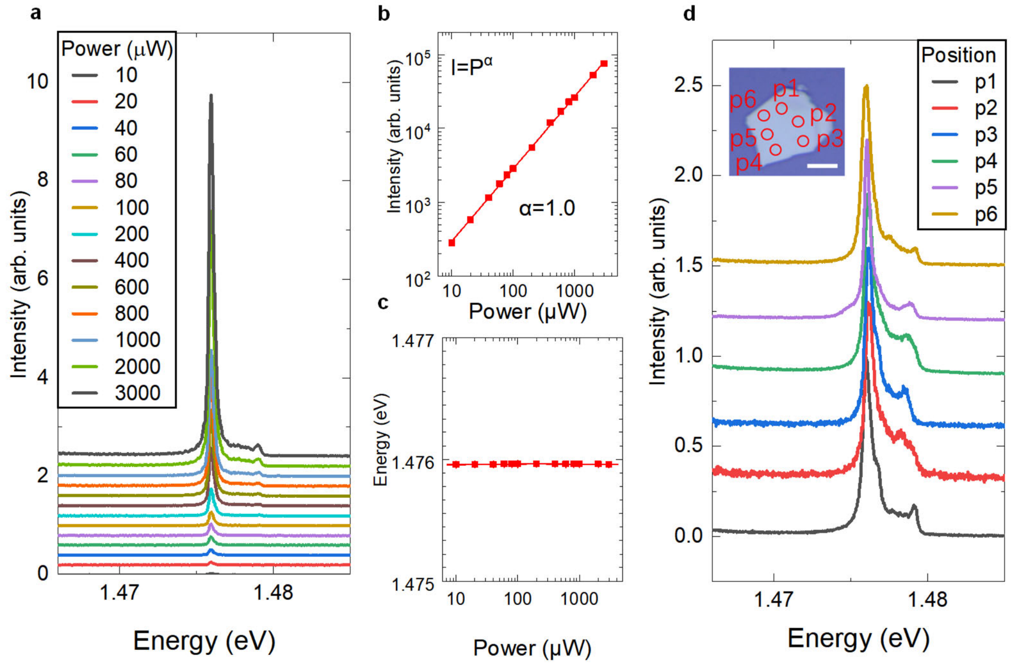

Supplementary Figure S3. Power-dependent and sample position-dependent PL spectra of ZRE. **a**, Power-dependent PL spectra of $NiPS_3$ thin flakes at 4 K. **b**, Excitation-power-dependent intensity of ZRE excited by a 633 nm laser. The slope of linear fitting indicates the linear dependence

of intensity on the excitation power in the range from 10 μW to 3 mW. **c**, Extracted peak position of ZRE as a function of the pump power. The error bars are defined as the fitting errors of the peak positions of ZRE in **a**. **d**, PL spectra of ZRE obtained from different positions of the same sample. Scale bar: 10 μm.

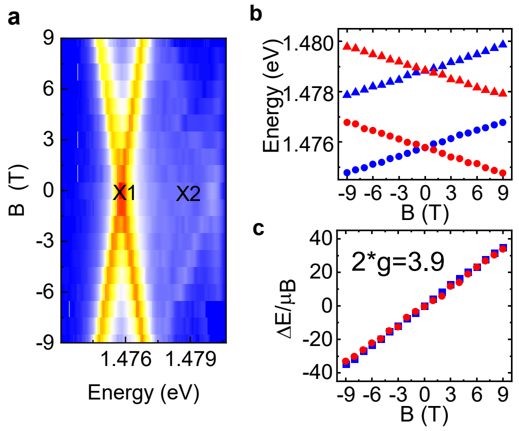

Supplementary Figure S4. Zeeman splitting of the spin-correlated excitons in sample_1. a, Contour plot of the PL spectra of the spin-correlated excitons in $NiPS_3$ as a function of the magnitude of the magnetic field, where the direction of the magnetic field is nearly parallel to the **a**-axis. **b**, The energy splitting of the X_1 and X_2 under the external magnetic field. The blue dots and red dots denote different splitting branches of the ZRE. The similar Zeeman splitting of X_1 and X_2 indicates that they are both coupled with the magnetic order. **c**, The effective *g*-factor of the X_1 is nearly the same as that of X_2 with a value of about 1.95, implying that the spin-orbital coupling has little influence on properties of electrons.

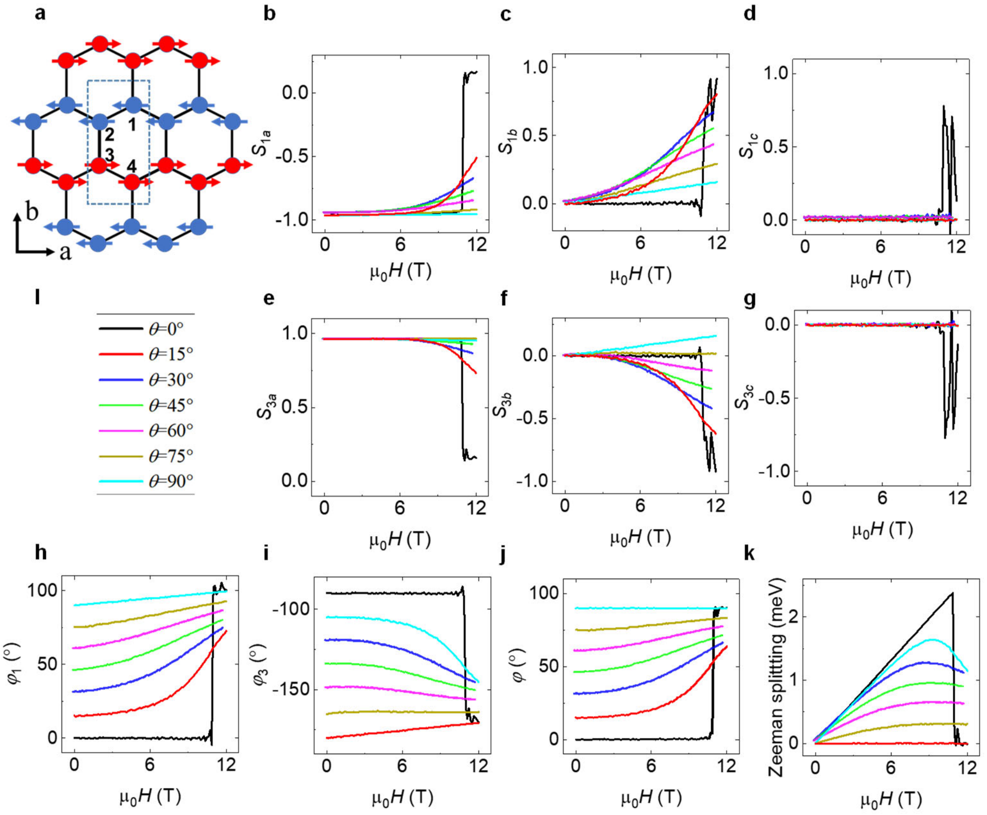

Supplementary Figure S5. Monte Carlo simulations of the spin orientation with 2D XY-type Heisenberg Hamiltonian. **a**, Crystal structure and unit cell used in Monte Carlo simulations. **b-g**, Components of spins along the *a*, *b* and *c*-axes under different magnetic fields, as calculated through Monte Carlo simulations with configurations performed according to the Method and **Supplementary note 3**. The $S_{1\alpha}$ represents the spin component along the **a**-axis of at position 1 in **a**, and so on. **h** and **i**, Magnetic field dependence of angles between the spin orientations at position 1 and 3 relative to the **a**-axis, extracted from panels **b-g. j**, Magnetic field dependence of the angles between the Néel vectors and *a*-axis extracted from panels **h** and **i. k**, Magnetic field dependence of the Zeeman splitting extracted from panels **g. l**, The plot legends of b-k.

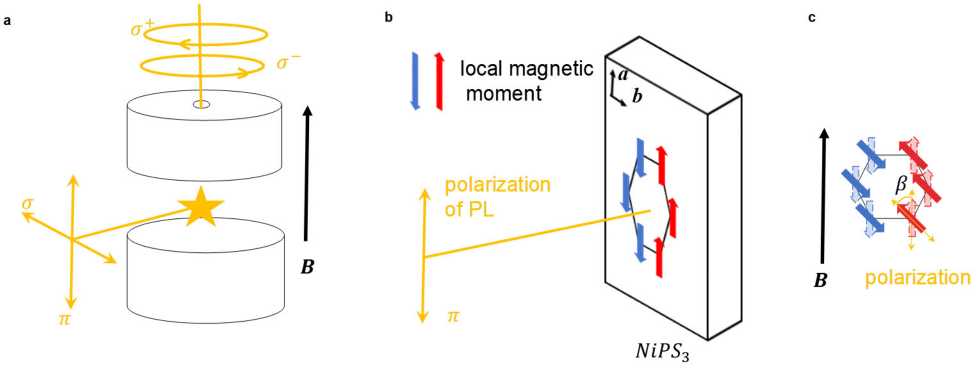

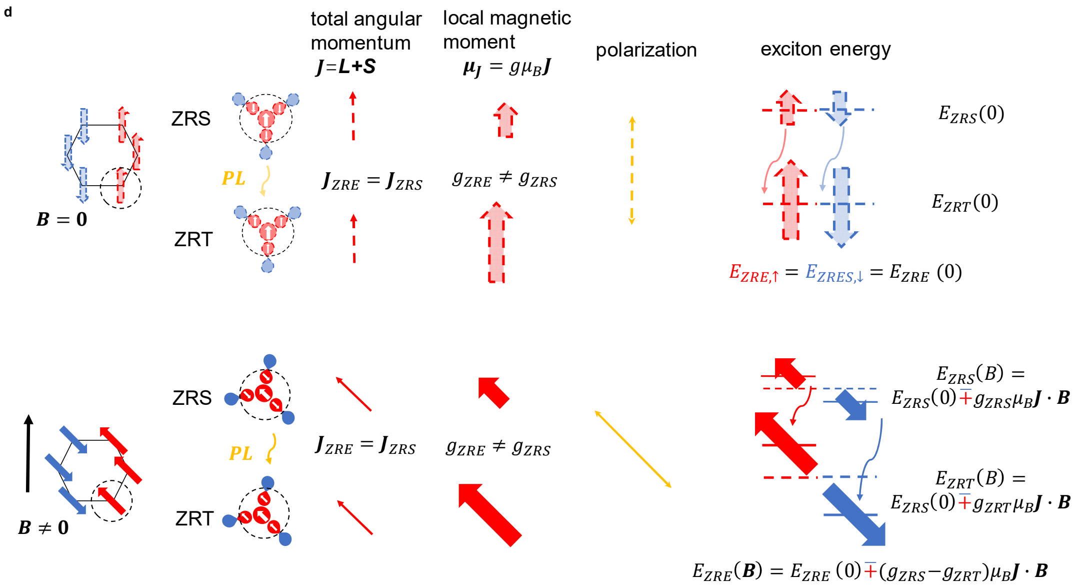

Supplementary Figure S6. Polarization, magnetic moment, and Zeeman effect of ZRE. a. Polarization of Zeeman effect in metal ions. **b.** Polarization of ZRE in our experiment. The blue and red arrow denote the local magnetic moment at Ni site in the lattice. **c.** Cartoon of the polarization rotation of ZRE. The β is defined as the rotation angle of polarization of ZRE with and without magnetic field. The blue, red, and black arrows denote the local magnetic moment at Ni site in the lattice, the polarization of the exciton localized around one Ni site, and the external magnetic field, respectively. The dash and solid arrow denote the condition with and without the external field. **d.** The transition between ZRT and ZRS under the external field.

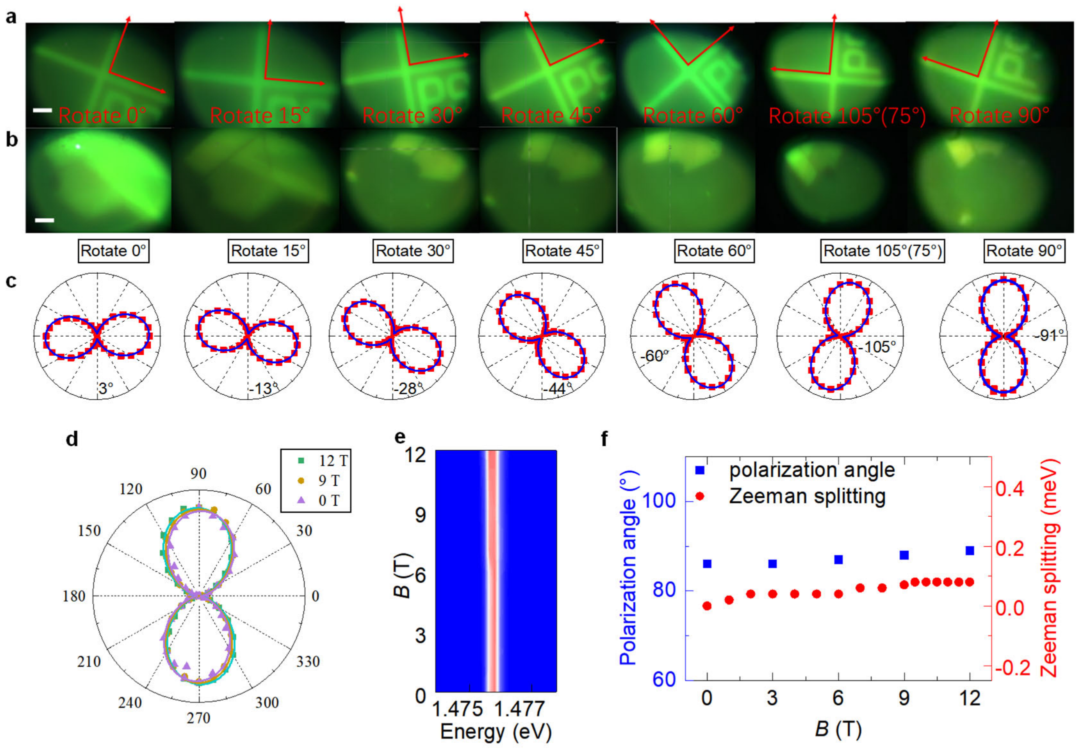

Supplementary Figure S7. Sample images and polarization-resolved PL of ZRE of sample_2

when the magnetic field is nearly along the b-axis. a, Real-time microscopic imaging of the gold marker on the substrate. From the left panel to the right panel, the gold marker was rotated by an in-situ rotator. Scale bar: 10 µm. **b**, Real-time microscopic imaging of the $NiPS_3$ thin flakes with different rotating angles. Scale bar: 10 µm. **c**, The polarization-resolved PL measurement of the $NiPS_3$ thin flakes with different rotating angles. With the real-time microscopic imaging, polarized PL measurement and Zeeman splitting, we can obtain an accurate angle of rotation with an error of no more than 3°. **d**, The polarization-resolved PL measurement when the magnetic field is nearly along the **b**-axis of the crystal with a magnetic field of 0 T, 9 T, 12 T, respectively. **e**, Contour plot of the PL spectra under different magnitude of the magnetic field. **f**, The polarization angles and Zeeman splitting as a function of *B*.

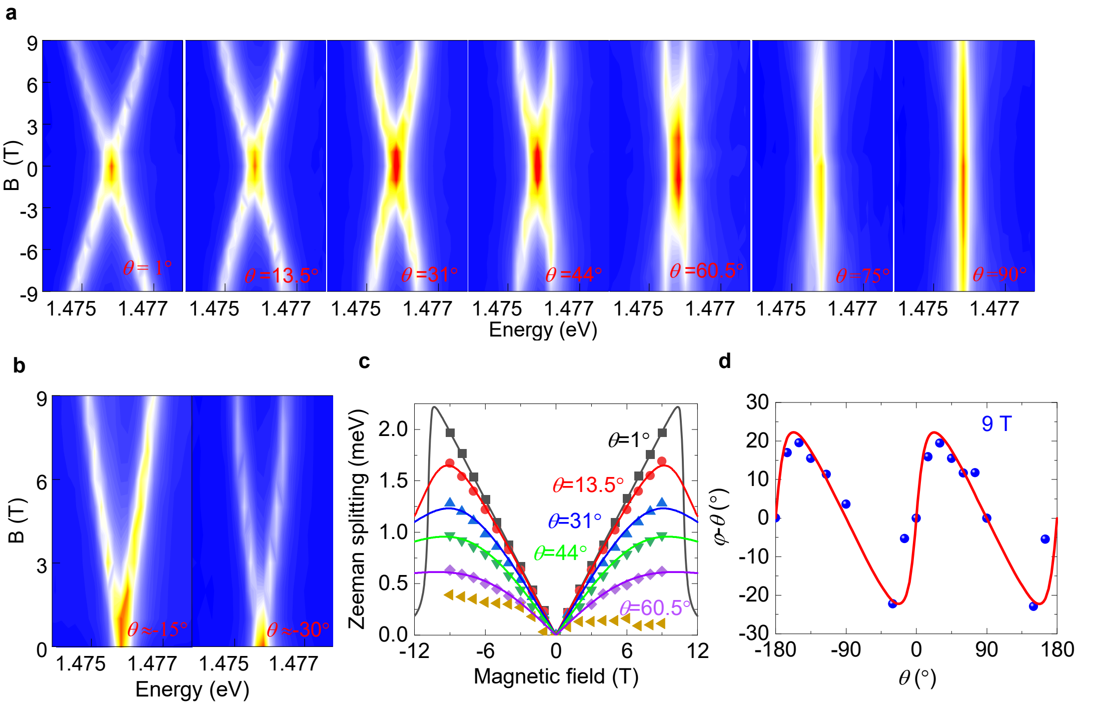

Supplementary Figure S8. The PL spectra of ZRE in sample_1 measured at 4 K with varying the magnetic field $\mu_0 H$. a and **b**, The contour plots of *B*-dependent PL spectra with different orientations of sample. **c**, The Zeeman splitting extracted from a and the corresponding fitting curves. The data is fitted with formula (S1) and (S2). **d**, The θ dependence of the rotation angle of the Néel vector ($\varphi - \theta$) under 9 T. The dots represent the data extracted from c and the line represents the fitting curve with formula (S1) and (S2).

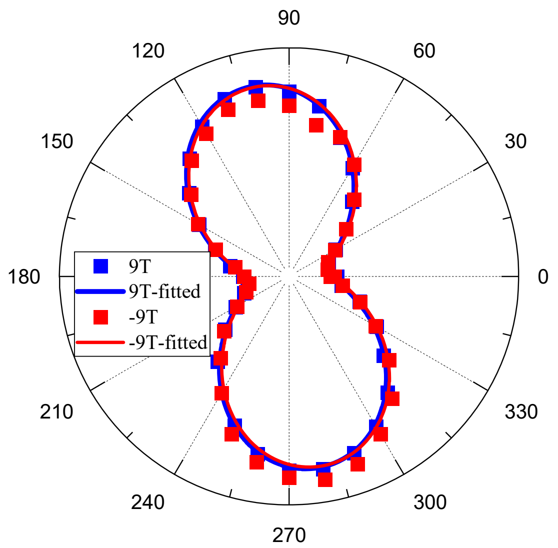

Supplementary Figure S9. Polarization-resolved PL intensity of ZRE under an external magnetic field perpendicular to a-axis. The red and blue solid squares are the experimental data of polarization at -9 T and 9 T, respectively, while the red and blue lines are the fitted curves with the sine function. The polarization of PL does not rotate when flipping the magnetic field, excluding the Faraday rotation effect in our experiment.

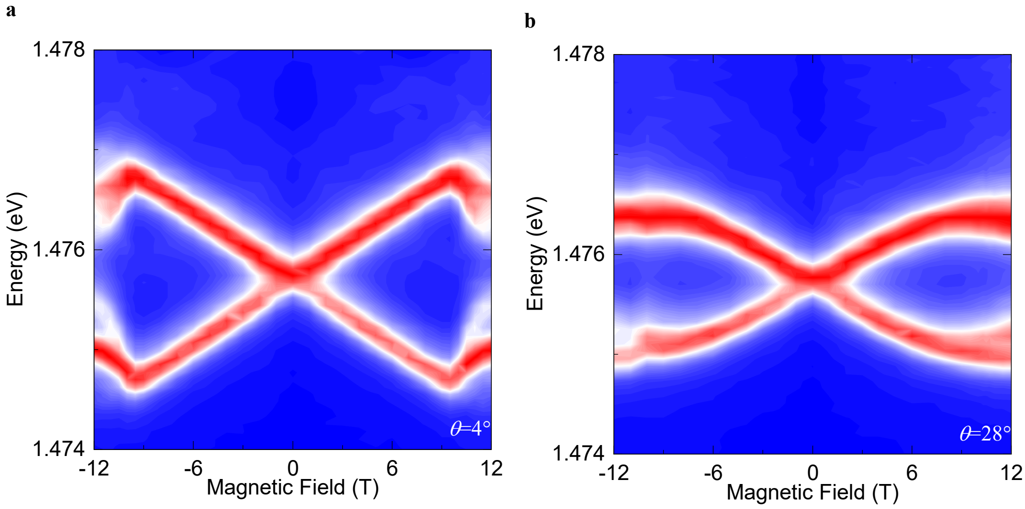

Supplementary Figure S10. The PL spectra of ZRE in sample_2 with flipping the magnetic field $\mu_0 H$. The SRT behaviors before and after flipping the magnetic field above 10 T are identical in **a**, $\theta = 4^{\circ}$, and **b**, $\theta = 28^{\circ}$, respectively.

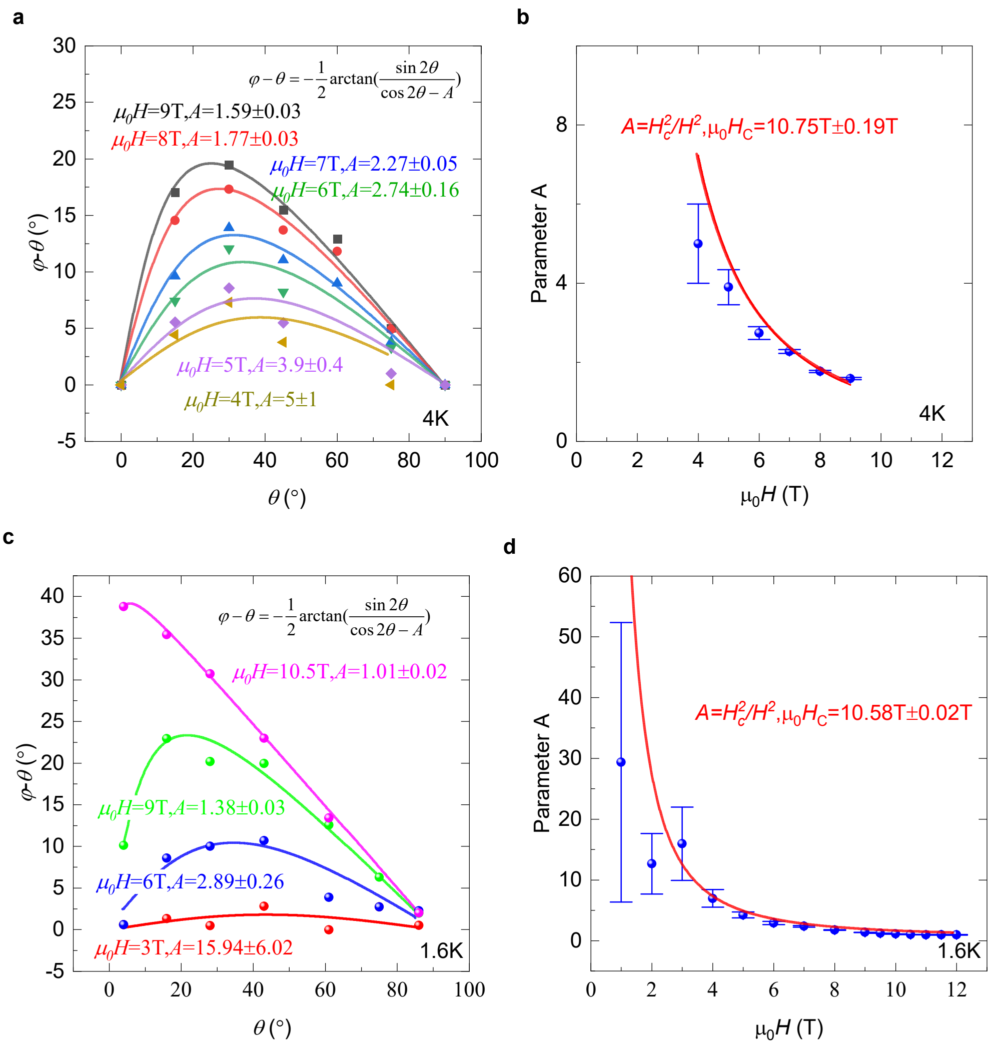

Supplementary Figure S11. Magnetic field-dependent parameter A of sample_1 at 4 K and sample_2 at 1.6 K. The PL spectra of sample_1 are shown in Supplementary Figure S6. The PL spectra of sample_2 are shown in Figure 3. **a**, θ dependence of $\varphi - \theta$ in sample_1 at different $\mu_0 H$. The data could be fitted by a formula with a parameter A, as shown in the figure. The dots represent the experimental data with different $\mu_0 H$. The lines represent the fit curves. The applied magnetic field and fit parameter are indicated on the figure. **b,** The dependence of A on $\mu_0 H$ of sample_1. The error bars are defined as the fitting errors of the A in **a**. The data could be fitted with the formula $A = \frac{H_C^2}{H^2}$. The red curve is the fitting curve. $\mu_0 H_c$ is about 10.75 T, in consistent with the reported result[20]. **c**, θ dependence of $\varphi - \theta$ in sample_2 under different $\mu_0\mathbf{H}$. **d**, The dependence of A on $\mu_0 H$ of sample_2. The error bars are defined as the fitting errors of the A in **c**.

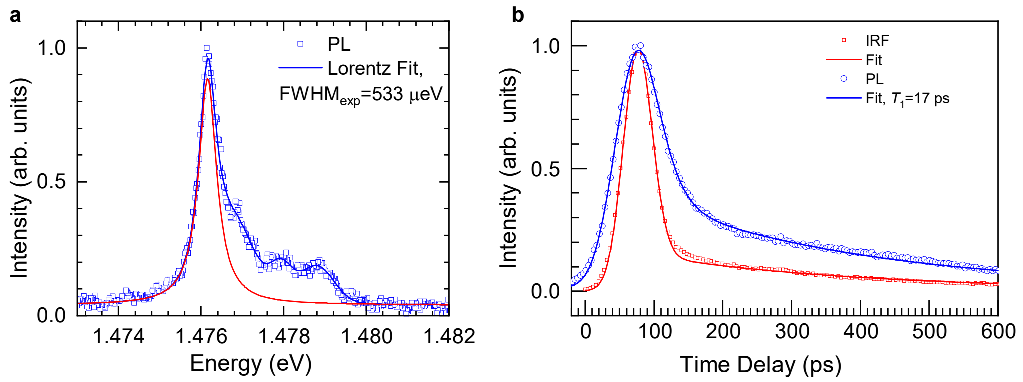

Supplementary Figure S12. The lifetime and linewidth of ZRE. a, The PL and its fitting of ZRE. **b**, The time-resolved PL spectrum of ZRE and its fitting. The time-resolved PL experiment was conducted using a time-correlated single photon counting technique and was excited by 440 nm pulse laser at 4 K. We obtain the linewidth (Γ) using the following equation: $FWHM_{exp} = \sqrt{\Gamma^2 + \Gamma_{inst}^2}$, where $FWHM_{exp}$ is the experimentally measured full-width-of-half-maximum of ZRE, Γ_{inst}^2 is instrument broadening, respectively. In our sample, a typical measured linewidth is $FWHM_{exp}$ = 533 μeV, Γ_{inst} is 226 μeV and we got Γ = 482 μeV. The narrowest linewidth we measured is 260 μeV and corresponding Γ = 128 μeV. As shown in **b**, we got the lifetime T_1 = 17 ps by deconvoluting the time-resolved PL spectra. The instrument response function (IRF) is 20 ps. In principle, our system has a time resolution of IRF/5.

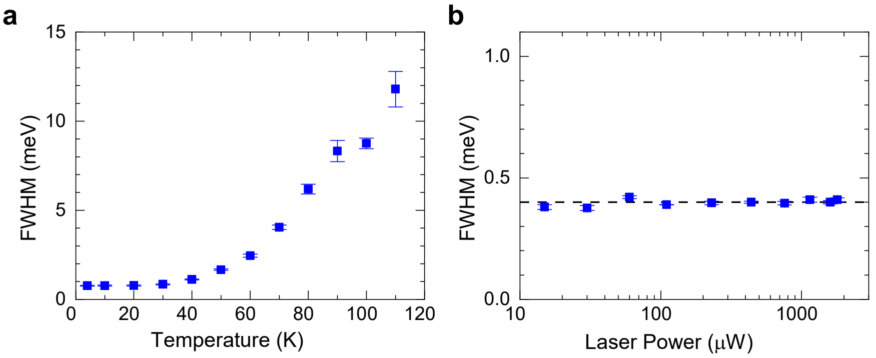

Supplementary Figure S13. The temperature and power dependence of ZRE FWHM. a, The temperature-dependent FWHM of ZRE, extracted from **Supplementary Figure S2 c**. **b**, The laser power-dependent FWHM of ZRE at 4K, extracted from **Supplementary Figure S3 d**.

	Lifetime (T_1)	$\hbar/2T_1$	Linewidth	Samples
Ref. 19	11 ps @ 5 K	30 μeV	350 μeV @ 10 K	CVT
Ref. 20	2~9 ps @ 10 K	36μeV~164 μeV	330 μeV @ 5 K	CVT
Ref. 21	40 ps @ 10 K	8 μeV	770 μeV @ 10 K	CVT
Ref. 18	~1 ns @ 4K	~0.3 μeV	1.7 meV @ 4 K	LQE

This work	17 ps @ 4K	19 μeV	482 μeV@ 4 K	CVT

Supplementary Table S1. The reports about the linewidth and lifetime of the ZRE. CVT means chemical vapor transport and LQE means liquid phase exfoliation.

Supplementary Reference

1 King, A. R. & Rohrer, H. The spinflop bicritical point in MnF_2. *AIP Conf. Proc.* **29**, 420-421 (1976).

2 Kim, K. *et al.* Suppression of magnetic ordering in XXZ-type antiferromagnetic monolayer $NiPS_3$. *Nat. Commun.* **10**, 345 (2019).

3 Andelkovic, Z. *et al.* Laser cooling of externally produced Mg ions in a Penning trap for sympathetic cooling of highly charged ions. *Phys. Rev. A* **87**, 033423 (2013).

4 Wang, G. *et al.* In-Plane Propagation of Light in Transition Metal Dichalcogenide Monolayers: Optical Selection Rules. *Phys. Rev. Lett.* **119**, 047401 (2017).

5 Kang, S. *et al.* Coherent many-body exciton in van der Waals antiferromagnet $NiPS_3$. *Nature* **583**, 785-789 (2020).

6 Klaproth, T. *et al.* Origin of the Magnetic Exciton in the van der Waals Antiferromagnet $NiPS_3$. *Phys. Rev. Lett.* **131**, 256504 (2023).

7 Lane, C. & Zhu, J.-X. Thickness dependence of electronic structure and optical properties of a correlated van der Waals antiferromagnetic $NiPS_3$ thin film. *Phys. Rev. B* **102** (2020).

8 Zhang, F. C. & Rice, T. M. Effective Hamiltonian for the superconducting Cu oxides. *Phys. Rev. B* **37**, 3759-3761 (1988).

9 Stöhr, J. & Siegmann, H. C. Magnetism. *Solid-State Sciences. Springer, Berlin, Heidelberg* **9**, 351-429 (2006).

10 Kudlacik, D. *et al.* Exciton and exciton-magnon photoluminescence in the antiferromagnet CuB_2O_4. *Phys. Rev. B* **102** (2020).

11 Sell, D. D., Greene, R. L. & White, R. M. Optical Exciton-Magnon Absorption in MnF_2. *Phys. Rev.* **158**, 489-510 (1967).

12 van der Ziel, J. P. Optical Spectrum of AntiferromagneticCr_2O_3. *Phys. Rev.* **161**, 483-492 (1967).

13 Mizushima, M. & Koide, S. ON THE LIFETIME OF THE LOWER TRIPLET STATES OF BENZENE. *J. Chem. Phys.* **20**, 765-769 (1952).

14 Stöhr, J. & Siegmann, H. C. Magnetism. *Solid-State Sciences. Springer, Berlin, Heidelberg* **11**, 479-520 (2006).

15 Cowan, R. D. *The theory of atomic structure and spectra*. (Univ of California Press, 1981).

16 Chai, R.-P., Kuang, X.-Y., Zhang, C.-X., Duan, M.-L. & Wang, H. Theoretical study of EPR spectra and local structure for (NiO6)10− cluster in LiNbO3:Ni2+ and Al2O3:Ni2+ systems. *Journal of Physics and Chemistry of Solids* **69**, 1848-1854 (2008).

17 Gerloch, M. & Miller, J. Covalence and the orbital reduction factor, k, in magnetochemistry. *Progress in Inorganic Chemistry*, 1-47 (1968).

18 Shcherbakov, A. *et al.* Solution-Processed NiPS3 Thin Films from Liquid Exfoliated Inks with Long-Lived Spin-Entangled Excitons. *ACS nano* **17**, 10423-10430 (2023).
19 Hwangbo, K. *et al.* Highly anisotropic excitons and multiple phonon bound states in a van der Waals antiferromagnetic insulator. *Nat. Nanotechnol.* **16**, 655-660 (2021).
20 Wang, X. *et al.* Spin-induced linear polarization of photoluminescence in antiferromagnetic van der Waals crystals. *Nat. Mater.* **20**, 964-970 (2021).
21 Li, Y., Liang, G., Kong, C., Sun, B. & Zhang, X. Charge-Transfer-Mediated Exciton Dynamics in Van der Waals Antiferromagnet NiPS3. *Advanced Functional Materials* **n/a**, 2402161
22 Stavrias, N. *et al.* Competition between homogeneous and inhomogeneous broadening of orbital transitions in Si:Bi. *Physical Review B* **96**, 155204 (2017).
23 Klingshirn, C. F. *Semiconductor optics*. Vol. 23 623-700 (Springer Science & Business Media, 2012).
24 Moody, G. *et al.* Intrinsic homogeneous linewidth and broadening mechanisms of excitons in monolayer transition metal dichalcogenides. *Nat Commun* **6**, 8315 (2015).
